# Identifying the Geographical Origin of Wolfberry Using Near-Infrared Spectroscopy and Stacking-Orthogonal Linear Discriminant Analysis

**DOI:** 10.3390/foods14101684

**Published:** 2025-05-09

**Authors:** Shijie Song, Xiaohong Wu, Mingyu Li, Bin Wu

**Affiliations:** 1 Mengxi Honors College, Jiangsu University, Zhenjiang 212013, China; 3220501008@stamail.ujs.edu.cn (S.S.); 3230501081@stamail.ujs.edu.cn (M.L.); 2 School of Electrical and Information Engineering, Jiangsu University, Zhenjiang 212013, China; 3 High-Tech Key Laboratory of Agricultural Equipment and Intelligence of Jiangsu Province, Jiangsu University, Zhenjiang 212013, China; 4 Department of Information Engineering, Chuzhou Polytechnic, Chuzhou 239000, China; 5 School of Computer Science and Engineering, Southeast University, Nanjing 211189, China

**Keywords:** wolfberry, origin identification, near-infrared spectroscopy, stacking ensemble learning

## Abstract

The geographical origin identification of wolfberry is key to ensuring its medicinal and edible quality. To accurately identify the geographical origin, the Stacking-Orthogonal Linear Discriminant Analysis (OLDA) algorithm was proposed by combining OLDA with the Stacking ensemble learning framework. In this study, Savitzky–Golay (SG) + Multiplicative Scatter Correction (MSC) served as the optimal preprocessing method. Four classifiers—K-Nearest Neighbors (KNN), Decision Tree, Support Vector Machine (SVM), and Naive Bayes—were used to explore 12 stacked combinations on 400 samples from five regions in Gansu: Zhangye, Yumen, Wuwei, Baiyin, and Dunhuang. When Principal Component Analysis (PCA), PCA + Linear Discriminant Analysis (LDA), and OLDA were used for feature extraction, Stacking-OLDA achieved the highest average identification accuracy of 99%. The overall accuracy of stacked combinations was generally higher than that of single-classifier models. This study also assessed the role of different classifiers in different combinations, finding that Stacking-OLDA combined with KNN as the meta-classifier achieved the highest accuracy. Experimental results demonstrate that Stacking-OLDA has excellent classification performance, providing an effective approach for the accurate classification of wolfberry origins and offering an innovative solution for quality control in the food industry.

## 1. Introduction

Wolfberry is both Chinese medicine and food, and it is a kind of medicine and homologous food [1,2]. It is also one of the 87 Chinese medicinal materials that can be used in ordinary and functional foods [3,4]. With the increasing recognition of foreign consumers for the nutrition and healthcare efficacy of wolfberry, the market for wolfberry is expanding, and the export volume of Chinese wolfberry is increasing year on year [5]. The origin of wolfberry has an important effect on its quality. Gansu is one of the main producing areas and its wolfberry is famous for its high medicinal value and nourishing effect. The traditional quality inspection of wolfberry is based on sensory identification, such as color, size, sweetness, and so on [5]. Some unscrupulous merchants may be shoddy or adulterated, infringing on the rights of consumers. For example, high-quality varieties such as ZhongNing wolfberries are in high demand. They were introduced to Jingyuan County, Baiyin City, and Gansu Province, but there are different varieties of wolfberries from other regions in Gansu Province or even Qinghai wolfberries in the markets to counterfeit and sell at a high price. In September 2024, China Central Television reported that sodium bisulfite was being excessively used in the production of wolfberries in some regions of Gansu Province to enhance their bright red color and boost their price. Therefore, it is necessary to develop an effective method for the classification of wolfberry origins to protect the rights and interests of consumers.

In recent years, the rapid development of Near-infrared Spectroscopy (NIRS) technology and machine learning algorithms has provided a new technological route for the identification of wolfberry origin. NIRS (wavelength range 780–2500 nm) mainly detects the vibration and rotation information of hydrogen-containing groups (such as C-H, O-H, N-H) in the molecules to obtain the chemical composition and structural characteristics of the sample. Since the main organic compounds in food (such as fats, proteins, and carbohydrates) contain these groups, NIRS can effectively reflect their content and structural characteristics [6,7]. NIRS analysis operates without chemical reagents, aligning with the principles of green detection [8]. Therefore, NIRS has gained recognition as an advanced analytical method in food processing, particularly due to its cost-effectiveness [9]. Machine learning algorithms can mine potential patterns from a large amount of spectral data, realizing accurate classification and prediction of samples. Therefore, for food qualitative research, an important direction is the combination of NIRS and machine learning algorithms for the classification and identification of food. For example, NIRS combined with chemometric tools was used to authenticate and classify plant-based protein powders using the One-Class Partial Least Squares (OC-PLS) model and Partial Least Squares Discriminant Analysis (PLS-DA) model [10]. Yin et al. investigated the feasibility of NIR and chemometrics for analyzing wolfberry samples from four different topographic regions in China. They combined near-infrared technology with chemometric methods, specifically Extreme Learning Machine models (ELM) based on feature wavelengths extracted from PCA loadings, for rapid geographic origin traceability of Chinese wolfberry (Goji) samples [11]. Similarly, Yahui et al. used near-infrared (NIR) spectroscopy combined with chemometric methods, particularly Least Squares Support Vector Machine (LS-SVM) for geographical origin identification and Synergy Interval Partial Least Squares (Si-PLS) for anthocyanin content prediction and rapid and effective determination of black wolfberry geographical origins and characteristic categories [12]. Nirere et al. (2022) further advanced this approach by incorporating Savitzky–Golay (SG) Smoothing and Standard Normal Variate (SNV) into the LS-SVM model, achieving a classification accuracy of 93.33% for dried wolfberry [13].

The above-mentioned studies mainly focus on the classification of wolfberry varieties in several major provinces of China, but even between different regions within one province, the quality of wolfberry varies significantly. Gansu Province in China is an important region for wolfberry cultivation. There are many important wolfberry-producing areas in Gansu Province, but there is relatively little research on the differences between wolfberries in these regions. Gansu Province is a significant player in China’s wolfberry market, and its produce is known for its high quality due to the favorable climate and soil conditions [14]. Therefore, the identification of the geographical origin of wolfberry is of great significance. In addition, although the above studies have established high-accuracy models through appropriate classifiers, they did not involve the effect of Stacking ensemble learning frameworks in wolfberry classification.

Ensemble techniques integrate multiple models to enhance predictive performance [15,16,17]. These approaches have been extensively applied across various research domains, including intelligent computation, and pattern recognition [18]. Moreover, ensemble strategies are widely recognized for their effectiveness in boosting the predictive capacity of machine learning models. Stacking, as one of the important methods of ensemble classification, has considerable research potential. Wolper first proposed the concept of Stacking generalization, discussing how to improve prediction performance by combining multiple different learners [19]. There are also several issues in the practical application of Stacking, such as the division of training data, and the interaction between base classifiers and meta-classifiers [20]. Džeroski et al. evaluated several state-of-the-art methods for constructing ensembles of heterogeneous classifiers with Stacking and showed that they performed (at best) comparably to selecting the best classifier from the ensemble method by cross-validation [21].

This study will classify the wolfberry origin from different regions of Gansu Province based on NIR and Stacking classification combined with different feature extraction methods including Principal Component Analysis (PCA), Linear Discriminant Analysis (LDA), and Orthogonal Linear Discriminant Analysis (OLDA). The principle of Stacking-PCA, Stacking-PCA + LDA, and Stacking-OLDA is to perform ensemble learning on feature data and use another meta-learner to learn how to combine the outputs of base models for final prediction. This study verifies the significance of the Stacking-OLDA model combined with NIRS for wolfberry origin identification.

## 2. Materials and Methods

### 2.1. Samples

**Figure 1:**
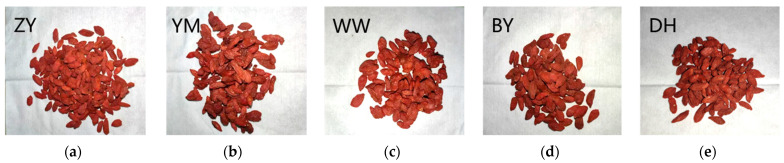
The wolfberry samples corresponding to five different geographical regions in Gansu Province. (**a**) Zhangye (ZY). (**b**) Yumen (YM). (**c**) Wuwei (WW). (**d**) Baiyin (BY). (**e**) Dunhuang (DH).

Four hundred dried wolfberry samples were selected and classified into five categories according to their origins. These samples were all from Gansu Province, the key production area of Chinese wolfberry. Each category consists of 80 samples from one region, specifically Zhangye (ZY), Yumen (YM), Wuwei (WW), Baiyin (BY), and Dunhuang (DH). Figure 1 shows wolfberries from different origins.

**Figure 2:**
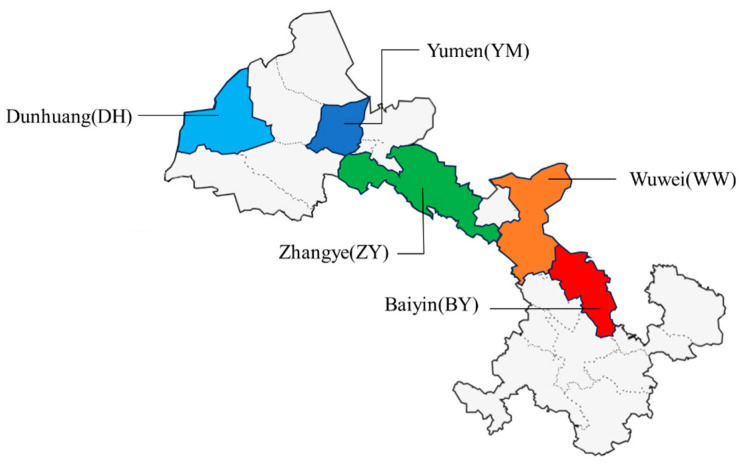
Geographical distribution of the wolfberry samples.

Since fresh goji berries are difficult to store and transport for a long time, most goji berries on the market are dried goji berries after drying treatment, and the mainstream drying treatment is hot air treatment [22]. The Goji berries selected in this study are hot air-dried products at the temperature of 50–60 °C. For each category, 80 samples were selected to ensure uniform size and good coloring. The dried fruit’s skin was intact without damage, and the fruit stems were attached. This was to ensure that no unnecessary nutrient loss occurred before the spectral data collection, with the fruit stems being removed before spectral analysis. The dried fruits were of uniform size and shape, without significant compression. During the selection process, the main characteristics of wolfberry from the same origin were observed, such as the typical size and color. Wolfberries with significantly different developmental stages within the same category were excluded, and samples that conformed to the general characteristics were chosen. Visually, dried wolfberries from ZhangYe appear smaller in size and more uniform in shape and wolfberries from Baiyin are generally more regular, showing full drops of water. Figure 2 illustrates the geographical distribution of the five different production regions of the Wolfberry samples.

Wolfberry is rich in a variety of organic compounds, including polysaccharides, amino acids, flavonoids, carotenoids, and fatty acids, and the molecular structure of these components contains a large number of hydrogen bonds (C-H, N-H, O-H) [23,24]. Gansu wolfberry is rich in a variety of nutrients and active ingredients. Lu, Y. et al. pointed out that BaiYin wolfberries in Gansu Province exhibited the highest total flavonoid content, which was higher than that of other producing areas [25]. The carotenoid content of BaiYin wolfberries ranked second among 13 major producing areas and the total phenolic content of ZhangYe wolfberries in Gansu Province ranked second among 13 major producing areas [25]. Since near-infrared (NIR) spectra primarily record the overtone and combination band absorption of the vibrational frequencies of hydrogen-containing functional groups, the chemical composition of wolfberry exhibits characteristic absorption features in NIR spectra. The NIR light band is mainly the frequency doubling and fusion absorption of the vibration of hydrogen-containing groups, which contains information on the composition and molecular structure of most types of organic compounds. The analysis in this study focuses on the spectral features within the 900.7592~1674.9758 nm range, where the absorption peaks associated with key chemical components like polysaccharides, moisture, and carotenoids are prominent. This range is also determined by the physical limitations of the spectrometer, which operates within a wavelength range of 900–1700 nm, thus restricting the measurement of other possible effective spectral data beyond this range. The absorption peaks of different chemical components in wolfberry correspond to different near-infrared wavelength ranges, as shown in Table 1.

The absorption peak below 1000 nm was attributed to the second vibration of N-H bonds in proteins or amino acids. At about 1200 nm, a relatively gentle absorption peak appeared due to the existence of wolfberry polysaccharide and flavonoid. At about 1450 nm, a more obvious absorption peak appeared due to the existence of moisture and phenol [11]. At approximately 1650 nm, tiny absorption peaks appeared due to the presence of carotenoids. The difference in the main chemical components mentioned above is the key to distinguishing different regions of Gansu wolfberry.

### 2.2. Spectra Acquisition

In this research, a fiber optic near-infrared (NIR) spectrometer NIR-M-F1-C (Shenzhen Pynect Science and Technology Co., Ltd., Shenzhen, China) was used to obtain spectral data of wolfberry samples. The near-infrared spectral module of this model is designed for grating splitting. It operates within a 900–1700 nm wavelength range, featuring a signal-to-noise ratio (SNR) of 6000:1 and a slit dimension of 1.8 × 0.025 mm. Its optical resolution averages 10 nm, with a peak resolution of 12 nm. Its wavelength accuracy typically falls within ±1 nm of the true value, with a maximum deviation of ±2 nm. The laboratory temperature was kept at 25 °C and the humidity was maintained at about 50% before the experiment. Dried wolfberry samples, along with the spectrometers, were stored in a well-ventilated and dry laboratory for over 24 h to ensure consistency in environmental conditions. This precaution was taken to reduce the potential impact of temperature and humidity fluctuations on spectral detection.

**Figure 3:**
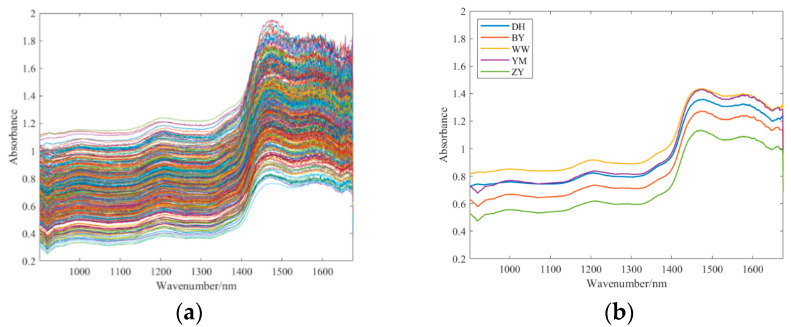
(**a**) The raw spectra of wolfberry. (**b**) The average spectra of each wolfberry category.

Before spectral acquisition, the spectrometer underwent a 30 min preheating process. After fixation, spectral data acquisition followed a columnar scanning framework, covering the 900–1700 nm spectral range. Each sample underwent three scans, and the resulting spectra were averaged to obtain a representative spectrum. NIR spectroscopy measurements were taken at equidistant points along the equatorial region of the wolfberry. For each sample, three NIR spectra were acquired, and their average was taken as the representative spectrum for that sample. A total of 80 samples of each type of wolfberry was measured, resulting in 400 near-infrared spectra. The effective wavelength range was determined to be 900.7592~1674.9758 nm. As shown in Figure 3, spectral absorbance increased significantly after 1400 nm and reached an absorption peak at 1440 nm.

In addition, the spectra within each wolfberry class were averaged to generate a reference spectrum for each wolfberry class, as shown in Figure 3b. The differences between YM and DH in the low band and between YM and WW in the high band are very small, reflecting that sample classification has considerable research value.

### 2.3. Spectral Preprocessing

Data preprocessing is of vital importance for data analysis [26,27]. Extracting effective features from raw spectra is crucial for spectral classification. NIR instruments inevitably produce measurement errors, noise and redundant information during the measurement process [28]. Consequently, raw spectra are susceptible to stochastic noise, baseline fluctuations, and light scattering effects. As shown in Figure 3a, the wavelength range after 1440 nm exhibits higher noise levels, making spectral preprocessing necessary. The most widely used spectral preprocessing methods include baseline correction, scattering correction and derivative [29].

#### 2.3.1. Baseline Correction

Baseline correction can improve the smoothness of NIR spectra. The Savitzky–Golay (SG) filter can be used to remove noise from spectral data [30]. SG smoothing is a well-established technique that utilizes polynomial functions to apply a least-squares fitting method within a specific window size [31]. More specifically, the SG filter performs localized polynomial regression to approximate data trends, ultimately refining the spectral profile. Additionally, taking the derivative of spectra using the SG often functions as an initial preprocessing approach to address overlapped spectral features, improve signal characteristics, and mitigate unwanted spectral variations caused by instrumental deviations and sample characteristics.

#### 2.3.2. Scatter Correction

Scattering correction can correct scattering effects and particle size effects, and two common methods are multiplicative scattering correction (MSC) and standard normal variable (SNV) [32]. MSC efficiently handles additive and multiplicative scattering effects by correcting for scattering effects by performing linear regression on each sample spectrum against the reference spectrum (usually the average spectrum) meaning that it depends on the entire sample set and is sensitive to outliers [33]. In contrast, SNV centers and standardizes each spectrum individually, does not require a reference spectrum, and thus does not depend on the sample set. It can handle new samples independently, is insensitive to outliers, mainly corrects for multiplicative scattering effects, and has relatively low computational complexity. The choice of these methods usually depends on the similarity of the samples, the stability of the dataset, and the specific application requirements. In general, MSC is suitable for processing cases with high sample similarity, while SNV is more suitable for handling cases with large sample differences.

#### 2.3.3. Derivative Spectra

The first derivative (FD) also plays an important role in spectral data preprocessing. By calculating the first derivative of the spectral data, the baseline drift can be effectively eliminated while improving the signal resolution [34]. Since FD emphasizes the rate of change in the signal, it is usually used to distinguish overlapped spectral peaks and reduce the errors caused by instrument and sample properties. In addition, FD can enhance the weak signal and make certain subtle peaks more obvious.

### 2.4. Principle Component Analysis

PCA, a generally utilized unsupervised machine learning technique [35], is employed to transform data into several significant components [36]. Its primary objective is to evaluate data covariance and change the dimensionality of the data from the high feature space to the low one. The range of information retained from the original data is related to the dimension of the new feature space, namely the selected principal component scores. Therefore, the principal component scores can significantly affect the prediction results of the prediction model [37].

**Figure 4:**
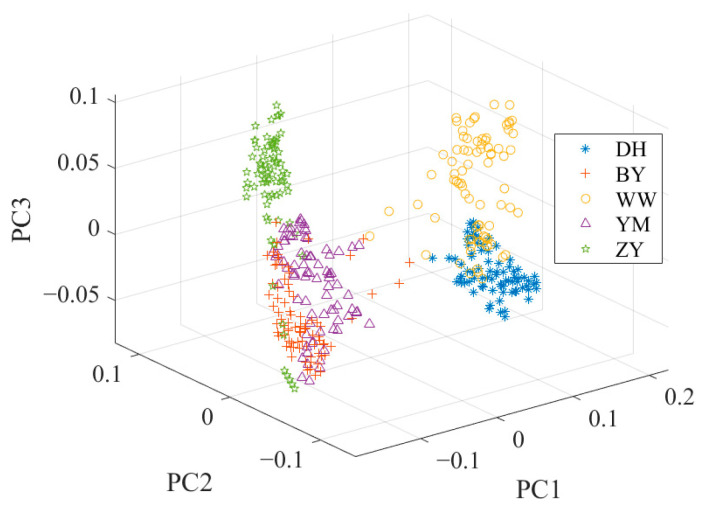
The data distribution after PCA.

The cumulative contribution rate of the first three principal components (PCs) amounted to 99.3%. Specifically, the first principal component (PC1) contributed 97.9%, while the second (PC2) and the third (PC3) accounted for 1.2% and 0.2%, respectively. Figure 4 shows the data distribution after PCA. Figure 4 also highlights a noticeable overlap between the spectral characteristics of BY and YM, potentially leading to a decrease in classification accuracy.

### 2.5. Linear Discriminant Analysis

LDA, a widely utilized supervised pattern recognition technique [38], reduces the dimensions of data by discriminant vectors and has been proven to be particularly effective in extracting data features. LDA can generally obtain more accurate classification results compared to PCA [39]. However, LDA suffers from certain constraints, especially when dealing with high-dimensional datasets with limited samples [40]. These constraints include the following aspects: first, LDA is prone to the “curse of dimensionality” when the dimensionality of data is much larger than the number of samples; second, when the class information is insufficient or the distribution among classes is uneven, the performance of LDA may be affected [41].

**Figure 5:**
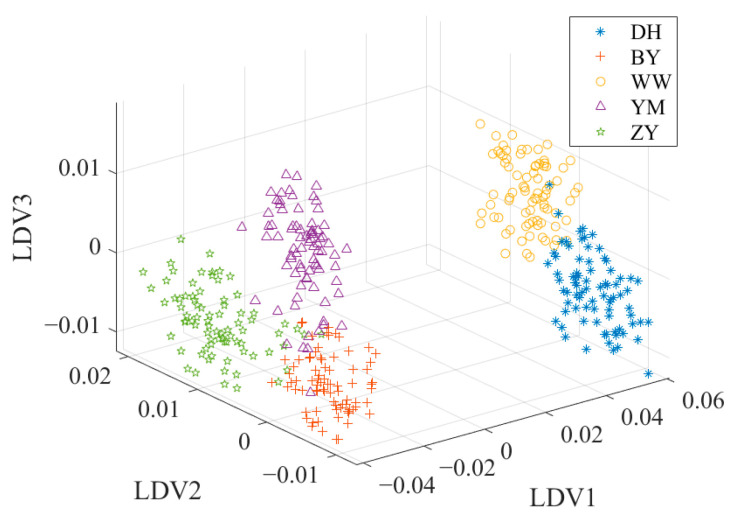
The data distribution after PCA + LDA.

To overcome these limitations, a combination of PCA and LDA is usually a solution to the problem. This combined framework usually consists of two key stages. In the PCA stage, the initial dimensionality reduction in the spectral data is performed to decrease the data dimensionality and decrease the noise effect. In the LDA stage, the discriminant information of the training data is used to map the test data to the discriminant vectors, and the secondary dimension reduction is realized to further enhance the classification ability of the data [42]. Figure 5 shows the data distribution after PCA + LDA. Compared with the data distribution after PCA in Figure 4, the overlap of the sample points in Figure 5 is significantly reduced. YM and BY have a better discrimination.

### 2.6. Orthogonal Linear Discriminant Analysis

**Figure 6:**
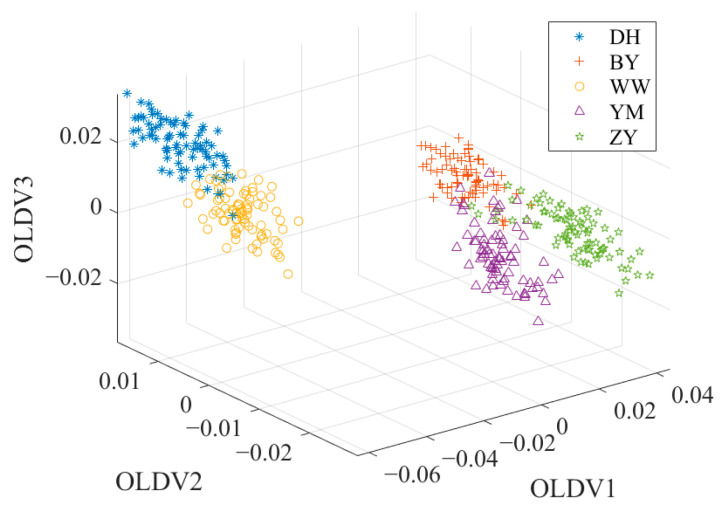
The data distribution after OLDA.

OLDA can overcome the singularity problem of the divergence matrix, which is common in high-dimensional and small-sample datasets. OLDA is an improved form of traditional LDA, which solves the key problem of multicollinearity between discriminant vectors [43]. Unlike traditional LDA, OLDA imposes an orthogonality constraint on the discriminant vectors, ensuring that the vectors are orthogonal to each other. This means that each discriminant vector is “independent” in the mathematical sense and they capture different aspects of the discriminant information in the data, rather than duplicate or similar information. Due to this orthogonality, OLDA can effectively improve the stability and robustness of classification performance while reducing redundancy. Therefore, OLDA is often used as one of the preferred methods for high-dimensional data classification in practical applications, such as gene expression data analysis, spectral data classification and other fields, and its ability has been widely recognized. Figure 6 shows the data distribution after OLDA. Compared with the data distribution after PCA or PCA + LDA, the sample points shown in Figure 6 have better discrimination. The OLDA algorithm is presented in reference [43].

### 2.7. Base Learners and Stacking Combinations

In the Stacking framework, base classifiers are first trained using the original training data, producing predictions that are referred to as meta-features. These meta-features are subsequently utilized to train a meta-learner. The effectiveness of Stacking relies on selecting base learners that are well suited to the specific problem. The meta-learner typically employs a technique with strong generalization capability to mitigate biases introduced by multiple base learners, enhancing robustness and reducing overfitting [44].

**Figure 7:**
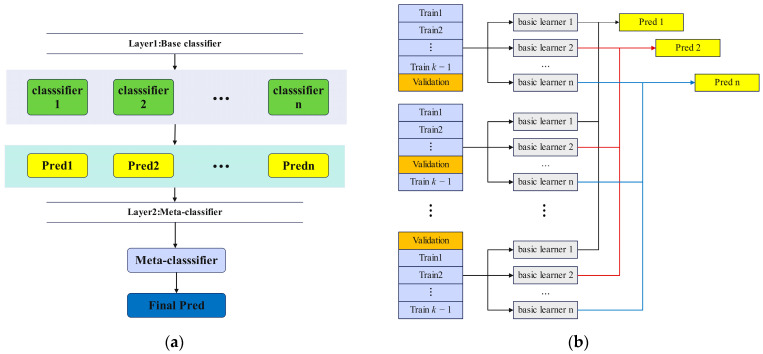
(**a**) Stacking ensemble learning framework. (**b**) Cross-validation strategy generates meta-features.

The framework, illustrated in Figure 7, involves constructing a meta-feature dataset through k-fold cross-validation. This approach ensures that every instance from the original training set contributes to generating meta-features, thereby enhancing model stability [45]. Additionally, k-fold cross-validation minimizes the risk of data leakage by partitioning the training set into k subsets. In each iteration, k − 1 subsets are used to train the base classifiers, while the remaining subset is reserved for meta-feature extraction. This procedure is repeated k times, ensuring comprehensive utilization of the dataset. Figure 7 shows the flowchart of k-fold cross-validation to generate meta-features.

In this study, KNN, Decision Tree, SVM, and Naive Bayes were selected for Stacking combination. The four classifiers have different learning mechanisms. Decision Tree is based on logical judgment; KNN is based on example learning of distance [46]; SVM is based on boundary division of hyperplane [47]; Naive Bayes is based on statistical judgment of probability. Such diversity is beneficial to the ensemble model to capture data characteristics from different angles and provide complementary prediction results.

#### 2.7.1. Two Base Classifiers and One Meta-Classifier

The simplest stacked framework contains two classes of base classifiers in the first layer and one meta-classifier in the second layer, that is, the ensemble classifier contains 3 different classifiers. It is worth noting that if Naive Bayes is served as a meta-classifier, there may be a problem that the input feature variance is 0 and the model cannot effectively estimate the distribution, resulting in a failed fit or a decrease in accuracy. This is because Naive Bayes assumes that the input features have a certain square, but when two or more base classifiers make the same prediction for the same y samples, these features received by Naive Bayes have zero variance in some classes and the model will fail. Therefore, the ensemble classifier with Naive Bayes as the meta-classifier is eliminated from the effective combination. Table 2 shows the combined classifiers and their serial numbers.

#### 2.7.2. Three Base Classifiers and One Meta-Classifier

In order to verify whether more weak classifiers can improve the classification performance of Stacking classifiers, this study also designed a Stacking framework with three base classifiers plus one meta-classifier. Since Naive Bayes cannot be used as a meta-classifier, there are three such combinations in total. Table 3 shows the combined classifiers and their numbers.

### 2.8. Comparing Other Ensemble Learning Frameworks

Compared to other ensemble learning methods used in previous research such as stacked ELM [11] and LS-SVM [12,13], the most significant innovation of the Stacking ensemble learning framework lies in its flexible heterogeneous integration capability and introduction of meta-learning mechanisms. Unlike ELM, which only integrates homogeneous fast neural network models, or LS-SVM, which relies on a single kernel function mapping strategy, Stacking combines multiple heterogeneous base models (such as KNN, SVM, Decision Trees, etc.) and further learns the correlations and optimal combination methods between models through a higher-order meta-classifier. This two-layer structure not only enhances the model’s expressiveness and generalization ability but also effectively reduces the risk of overfitting, making it particularly suitable for classification problems with complex features and significant performance variations across different models.

### 2.9. Software

All the algorithms were programmed and performed by Matlab 2020b (The MathWorks, Natick, MA, USA).

## 3. Results

### 3.1. Preprocessing Results

**Figure 8:**
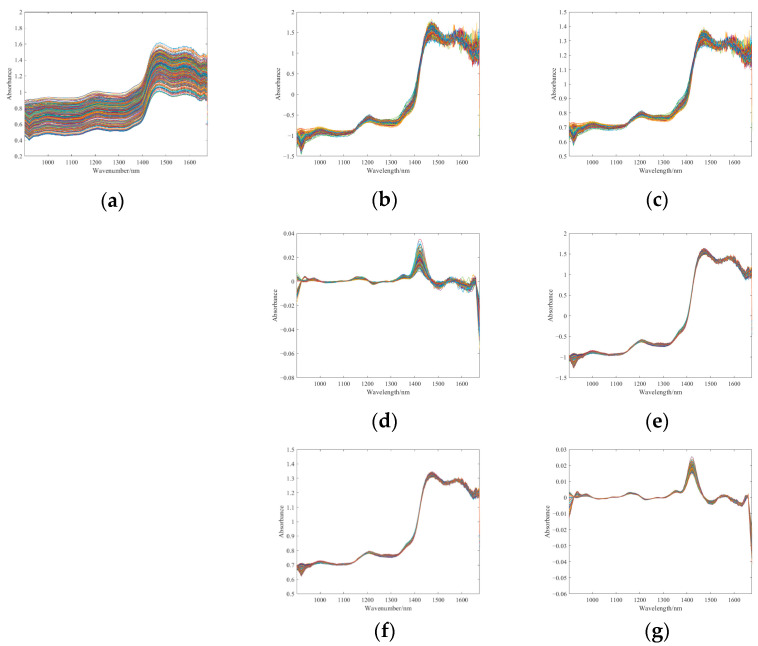
NIR spectra processed by different preprocessing methods. (**a**) SG. (**b**) SNV. (**c**) MSC. (**d**) FD. (**e**) SG and SNV. (**f**) SG and MSC. (**g**) SG and FD.

In this study, SG smoothing was performed on the raw spectra using a window size of 9 and a polynomial order of 1. The first derivative spectra were computed with a polynomial order of 1 and a window size of 9. Figure 8 shows spectra processed by different preprocessing methods.

The basic model of PCA + KNN was operated to evaluate how the data preprocessing techniques affect model performance. Five-fold cross-validation was employed. The average classification accuracy of different preprocessing methods under this test is shown in Table 4. After preprocessing, the classification accuracy was improved to varying degrees, and SG performed significantly better in a single preprocessing. By combining SG with other preprocessing methods, the classification accuracy was further improved, and the combination of SG + MSC had the highest classification accuracy, so it was applied as the specified method for subsequent preprocessing.

### 3.2. Stacking Classification Results and Optimization

#### 3.2.1. Classification Accuracy of Different Methods

To obtain reliable classification performance for each model and to clearly distinguish the accuracy rates of different classification methods, the accuracy of each model was calculated 10 times and averaged.

**Figure 9:**
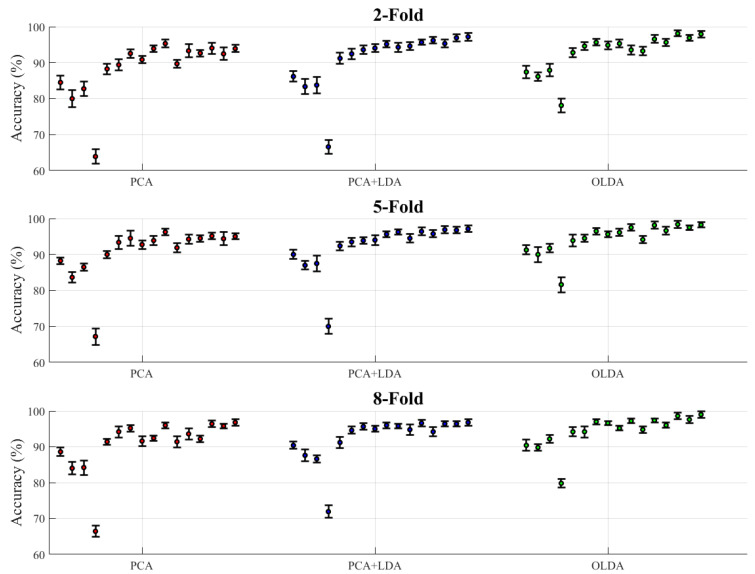
Accuracy of classification methods under different cross-validation settings.

Table 5 shows the classification accuracy of all classification methods under different k-fold cross-validation settings, and the classifiers were divided into two categories overall: a single classifier and Stacking ensemble classifiers. As can be seen from Table 5, the accuracy of the model including OLDA was overall better than other feature extraction algorithms. Figure 9 represents the average accuracy data in Table 5 as a scatter plot with error bars, the type of which is the standard deviation of each ten repeated experiments. The accuracy data under different k-folds were summarized to the corresponding feature extraction algorithm. The accuracy scatter under each feature extraction algorithm from left to right corresponds to the data from top to bottom of Table 5.

Moreover, when examining the k-fold cross-validation setting with k = 2, 5, 8, we observed some patterns across all three feature extraction methods. For the single classifier, increasing the number of folds generally led to a slight increase in accuracy, but the effect stabilized between 5-fold and 8-fold validations. The Stacking combination showed a more complex pattern under different k-fold settings. At Stacking 12, accuracy was 99% for OLDA under 8-fold validation, compared to 97.95% for 2-fold validation, which proved to be a meaningful improvement that validated the possible positive effect of choosing a higher value of k. However, it was also observed that the accuracy was not improved with the increase in the k value in some combinations, which might have been because the number of samples contained in the generated test set decreased with the increase in the k value, leading to the increase in the variance of the training results and the decline of the classifier performance.

Notably, the performance advantage of OLDA over PCA and PCA + LDA was maintained in all k-fold settings, indicating that its superior feature extraction capability was robust to different cross-validation strategies.

#### 3.2.2. Analysis of Classifiers’ Performance

This study further analyzed the effect of four weak classifiers when they served as base and meta-classifiers, respectively. The base classifier group included all four types of classifiers, while the meta-classifier contained KNN, Decision Tree, and SVM, excluding Naive Bayes.

**Figure 10:**
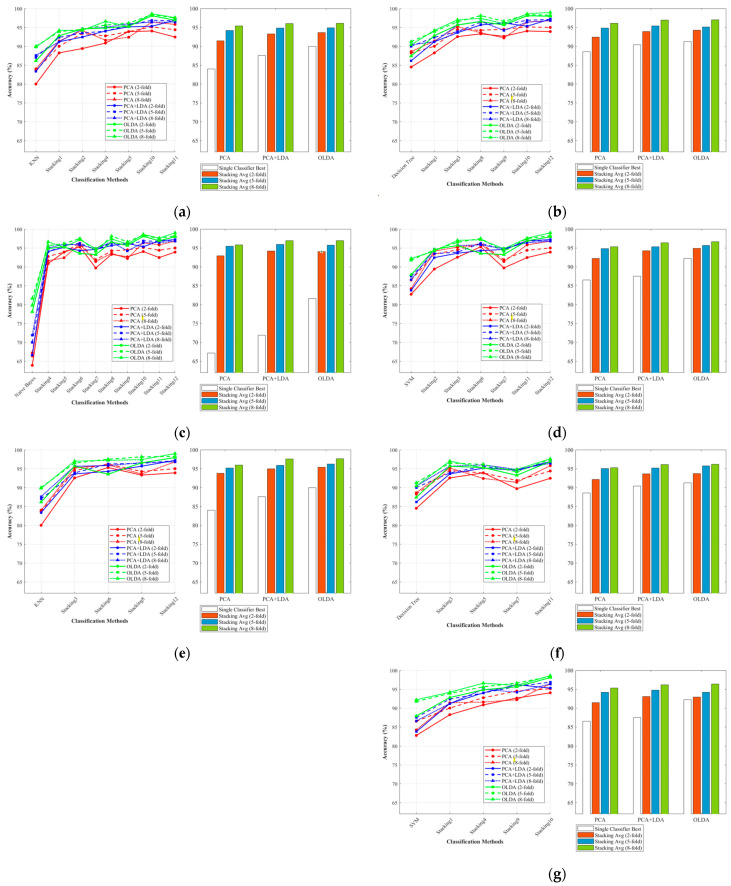
The average classification accuracies of Stacking combinations and different feature extraction methods with distinct base classifiers and meta-classifiers. (**a**) Base-KNN; (**b**) Base-TREE; (**c**) Base-NB; (**d**) Base-SVM; (**e**) Meta-KNN; (**f**) Meta-TREE; (**g**) Meta-SVM.

Figure 10a–d illustrate the average accuracy of the Stacking combination when KNN, Decision Tree, Naive Bayes, and SVM were performed as base classifiers, respectively. Figure 10e–g show the corresponding results of KNN, Decision Tree, and SVM as meta-classifiers. Each figure was composed of two subplots, the line chart on the left captures the required classification methods, and the bar chart on the right was divided into three clusters with four columns each. The white columns showed the highest accuracy of a single classifier in the 2-fold, 5-fold, and 8-fold settings for the specified feature extraction algorithm. The remaining three columns represented the average accuracy of the Stacking classifier for the three folds, respectively. The results intuitively showed that the overall accuracy increased from 2-fold to 8-fold. It was worth noting that Naive Bayes performed extremely poorly when used as a single classifier, but when used as a base classifier, it did not perform as badly as when used as a single classifier. The results showed that Stacking’s ensemble learning framework weakened the negative performance of weak classifiers to some extent and effectively enhanced the overall performance of the combined classifier. It has been shown that models with KNN as the base classifier did not perform well (the highest average accuracy achieved by the Stacking-OLDA combination was 96.07% under the 8-fold setting).

However, when KNN was the meta-classifier, the model achieved significantly higher accuracy compared with other meta-classifiers. It achieved the highest average accuracy (95.4%, 96.21%, and 97.65%, respectively) on three models (Stacking-PCA, Stacking-PCA + LDA, Stacking-OLDA).

**Figure 11:**
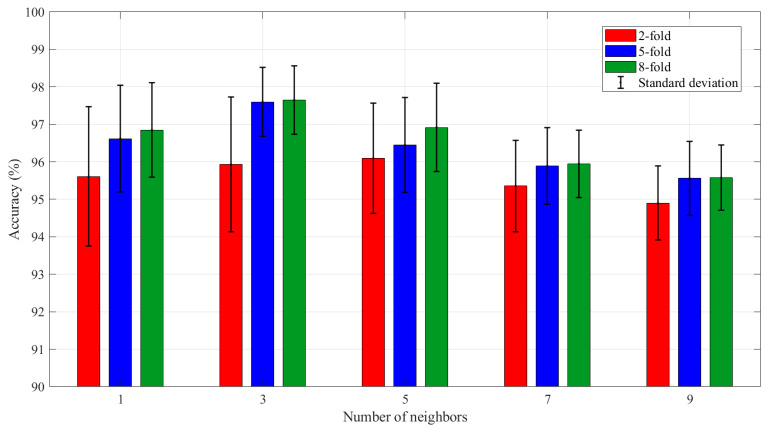
The average accuracy of Stacking-OLDA for different numbers of neighbors.

In order to further optimize the performance of KNN as a meta-classifier in the Stacking-OLDA model, the hyperparameters of KNN were adjusted by testing different neighbor counts (K = 1, 3, 5, 7, 9). As shown in Figure 11, when K = 3, the 5-fold model achieved the highest average accuracy of 97.60% and the 8-fold model achieved the highest average accuracy of 97.65%. However, the highest accuracy rate of the 2-fold model occurred when K = 5, and the accuracy rate at this time was 96.10%.

## 4. Conclusions

In this study, a Stacking ensemble classifier framework combining multiple feature extraction methods was proposed to classify the origin of wolfberry in Gansu Province, China. The Stacking-OLDA model was established to verify the feasibility and effectiveness of the Stacking model in this task. The dataset included 400 samples of wolfberry collected using Near-infrared Spectroscopy (NIRS) from five different producing areas in Gansu Province. Firstly, preprocessing methods including SG, SNV, MSC, and FD were evaluated, and SG + MSC was selected as the optimal preprocessing method. Secondly, 12 Stacking combinations were constructed, and the classifiers were trained using cross-validation with 2-fold, 5-fold, and 8-fold settings. Then, three feature extraction methods—PCA, PCA + LDA, and OLDA—were tested. The results showed that the classification accuracy of the Stacking-OLDA model was superior to that of the Stacking-PCA and Stacking-PCA + LDA models, with the highest average accuracy of 99% in 8-fold testing. It was also found that increasing the number of base classifiers further improved the model’s performance. Notably, the Stacking learning framework achieved the best average performance when KNN served as the meta-classifier. These results confirmed that the Stacking-OLDA model demonstrated excellent classification accuracy and provided valuable insights for the future development of Stacking ensemble classification models with more base classifiers.

This study has certain limitations that should be acknowledged. One, the dataset was collected from a single laboratory, which may limit the generalizability of the model to other environmental variations. The other was that while 8-fold cross-validation was used to evaluate model performance, the higher-fold cross-validation may increase the risk of overfitting due to smaller validation subsets and potentially unstable meta-feature generation in the Stacking framework. To address this, future work will consider repeated cross-validation, external test sets, and domain adaptation techniques to mitigate overfitting and enhance the reliability of the results in practical applications.

## Figures and Tables

**Table 1:** The absorption peaks of different chemical components in Gansu wolfberries.

Chemical Composition	Main Structural Group	Main Absorption Wavelength (nm)	Vibration Mode
protein/amino acid	N-H	970–1050	secondary stretching vibration
wolfberry polysaccharide	C-H, O-H	1150–1250	secondary stretching vibration
flavonoid	C-H	1200	secondary stretching vibration
phenol	O-H	1400–1450	first overtone of stretching vibration
moisture	O-H	1400–1500	first overtone of stretching vibration
carotenoids	C-H	1640–1680	first overtone of stretching vibration

**Table 2:** Stacking ensemble classifiers consisting of two base classifiers and one meta-classifier.

Methods	Base Classifier 1	Base Classifier 2	Meta Classifier
Stacking 1	KNN	Decision Tree	SVM
Stacking 2	KNN	SVM	Decision Tree
Stacking 3	SVM	Decision Tree	KNN
Stacking 4	KNN	Naive Bayes	SVM
Stacking 5	KNN	Naive Bayes	Decision Tree
Stacking 6	SVM	Naive Bayes	KNN
Stacking 7	SVM	Naive Bayes	Decision Tree
Stacking 8	Decision Tree	Naive Bayes	KNN
Stacking 9	Decision Tree	Naive Bayes	SVM

**Table 3:** Stacking ensemble classifiers consisting of three base classifiers and one meta-classifier.

Methods	Base Classifier 1	Base Classifier 2	Base Classifier 3	Meta Classifier
Stacking 10	KNN	Naive Bayes	Decision Tree	SVM
Stacking 11	KNN	Naive Bayes	SVM	Decision Tree
Stacking 12	SVM	Naive Bayes	Decision Tree	KNN

**Table 4:** Classification accuracy of PCA + KNN on the test set with different preprocessing methods.

Methods	Accuracy (%)	Methods	Accuracy (%)
RAW	44.1	FD	55.9
SG	83.6	SG + MSC	88.3
SNV	56.7	SG + SNV	86.0
MSC	59.3	SG + FD	85.5

**Table 5:** Accuracy of classification methods under different cross-validation settings.

Methods	PCA			PCA + LDA			OLDA		
	2-Fold	5-Fold	8-Fold	2-Fold	5-Fold	8-Fold	2-Fold	5-Fold	8-Fold
Single classifier									
KNN	84.50	88.25	88.60	86.15	90.00	90.40	87.40	91.25	90.40
Tree	80.00	83.63	84.00	83.35	87.00	87.60	86.15	90.00	89.80
SVM	82.75	86.50	84.20	83.75	87.50	86.60	87.90	91.75	92.20
Naive Bayes	63.90	67.20	66.40	66.60	70.00	71.90	78.10	81.63	79.80
Stacking combination									
Stacking 1	88.25	90.00	91.40	91.20	92.38	91.20	92.80	93.88	94.20
Stacking 2	89.40	93.38	94.20	92.45	93.50	92.80	94.60	94.50	94.20
Stacking 3	92.55	94.50	95.20	93.60	93.88	94.60	95.65	96.50	97.00
Stacking 4	90.85	92.75	91.60	94.05	94.00	95.00	94.85	95.63	96.60
Stacking 5	93.90	93.88	92.40	95.15	95.63	96.00	95.30	96.13	95.20
Stacking 6	95.30	96.25	96.00	94.30	96.25	95.80	93.55	97.50	97.20
Stacking 7	89.70	91.88	91.40	94.60	94.50	94.80	93.25	94.13	94.80
Stacking 8	93.30	94.25	93.60	95.65	96.40	96.60	96.55	98.13	97.40
Stacking 9	92.65	94.50	92.20	96.20	95.75	94.20	95.65	96.63	96.00
Stacking 10	94.05	95.13	96.40	95.30	96.88	96.40	98.10	98.38	98.60
Stacking 11	92.45	94.38	95.80	96.90	96.75	96.40	96.90	97.50	97.60
Stacking 12	93.90	95.00	96.80	97.20	97.13	96.80	97.95	98.25	99.00

## Data Availability

The original contributions presented in this study are included in the article. Further inquiries can be directed to the corresponding authors.
